# Solving global shallow water equations on heterogeneous supercomputers

**DOI:** 10.1371/journal.pone.0172583

**Published:** 2017-03-10

**Authors:** Haohuan Fu, Lin Gan, Chao Yang, Wei Xue, Lanning Wang, Xinliang Wang, Xiaomeng Huang, Guangwen Yang

**Affiliations:** 1 Ministry of Education Key Laboratory for Earth System Modeling, and Department of Earth System Science, Tsinghua University, Beijing, China; 2 Joint Center for Global Change Studies (JCGCS), Beijing, China; 3 Department of Computer Science and Technology, Tsinghua University, Beijing, China; 4 Institute of Software, Chinese Academy of Science, Beijing, China; 5 College of Global Change and Earth System Science, Beijing Normal University, Beijing, China; 6 National Supercomputing Center in Wuxi, Wuxi, China; Universidade de Vigo, SPAIN

## Abstract

The scientific demand for more accurate modeling of the climate system calls for more computing power to support higher resolutions, inclusion of more component models, more complicated physics schemes, and larger ensembles. As the recent improvements in computing power mostly come from the increasing number of nodes in a system and the integration of heterogeneous accelerators, how to scale the computing problems onto more nodes and various kinds of accelerators has become a challenge for the model development. This paper describes our efforts on developing a highly scalable framework for performing global atmospheric modeling on heterogeneous supercomputers equipped with various accelerators, such as GPU (Graphic Processing Unit), MIC (Many Integrated Core), and FPGA (Field Programmable Gate Arrays) cards. We propose a generalized partition scheme of the problem domain, so as to keep a balanced utilization of both CPU resources and accelerator resources. With optimizations on both computing and memory access patterns, we manage to achieve around 8 to 20 times speedup when comparing one hybrid GPU or MIC node with one CPU node with 12 cores. Using a customized FPGA-based data-flow engines, we see the potential to gain another 5 to 8 times improvement on performance. On heterogeneous supercomputers, such as Tianhe-1A and Tianhe-2, our framework is capable of achieving ideally linear scaling efficiency, and sustained double-precision performances of 581 Tflops on Tianhe-1A (using 3750 nodes) and 3.74 Pflops on Tianhe-2 (using 8644 nodes). Our study also provides an evaluation on the programming paradigm of various accelerator architectures (GPU, MIC, FPGA) for performing global atmospheric simulation, to form a picture about both the potential performance benefits and the programming efforts involved.

## Introduction

As one of the traditional high-performance computing (HPC) applications, atmospheric models have been one of the major consumers of supercomputer computing cycles [1] and major drivers of new HPC technologies [2–4].

Especially in recent years, the more and more urgent economic and social challenges brought by global warming are asking for better scientific understanding of the climate change mechanism and more accurate climate models to make predictions into the future climate risks. These demands, along with the inherent development of climate science, are calling for significantly more computing power, to support higher modeling resolutions [5], to include descriptions of more complex physics processes [6], and to enable more accurate modeling through larger ensembles [7].

With the demand for computing power constantly increasing, the computing resources that climate scientists are facing are also changing rapidly. While previous climate models are mostly based on CPU-only clusters, the large-scale supercomputers that are built these years are mostly employing heterogeneous systems that rely on accelerators, such as GPUs [8], MICs [9], or even reconfigurable FPGAs [10]. While these hybrid systems are providing more computing power, the inclusion of different architectures within one system is leading to more complicated programming patterns, and huge challenges on porting existing climate model softwares.

Considering the evolvements from both fronts, we see the toughest challenge as how to make the development trends of both domains meet, and make efficient utilization of emerging computing technologies for the scientific demands of climate models. In this paper, we present our initial efforts towards this long-term goal, describe our early experience on performing highly scalable and highly efficient atmospheric simulations on CPU-GPU (Tianhe-1A), CPU-MIC (Tianhe-2), and CPU-FPGA (Maxeler DFE), and provide our discussion across these different architectures and their corresponding programming paradigms.

Our major contributions and findings are as follows:

- a generalized partition scheme to achieve balanced utilization of both CPU resources and accelerator resources;
- optimization techniques based on many-core architectures (GPU, MIC) and customizable hardware accelerators (FPGA) to achieve one to two orders of magnitude per-socket speedup for solving the shallow water equations;
- close to linear scalabilities in both weak and strong scaling experiments on Tianhe-1A and Tianhe-2 supercomputers;
- through an extensive exploration on various architectures and their corresponding programming paradigms, we think that the accelerators demonstrate promising performance potentials, while the programming support is also close to a stage that can actively involve domain experts.

Compared with other HPC applications, the porting of the climate models onto new architectures has been largely constrained by the heavy legacies (millions of lines of code that evolves from a few decades before). When GPGPUs were firstly introduced to the scientific computing community, most efforts on utilizing accelerators in climate models were focused on the standalone physics modules. The Weather Research and Forecast (WRF) model, which is one of the most widely used numerical weather prediction (NWP) programs in the world, was among one of the earliest weather/climate models that integrate GPU-accelerated microphysics schemes [4]. Since then, we have observed similar efforts that port portions of weather or climate models to GPU platforms, such as the chemical kinetics modules in WRF-Chem [11], the shortwave radiation parameterization in the Community Atmospheric Model (CAM) [12], the cloud microphysics scheme in WRF [13], and the Global/Regional Assimilation and Prediction System (GRAPES) [14]. Due to the high arithmetic density in these physics modules, we typically see a speedup of one to two orders of magnitude (details shown in Table 1).

**Table 1:** Existing efforts on utilizing accelerators in climate models.

	Original	Acceleration	Original Unit	Acceleration Unit	Speedup
**Subroutine & Physics Core**					
**WRF WSM5** [4]	1 CPU (2.4GHz Dual-Core Opteron)	1.35 GHz Quadro 5600	0.33GFlops	15.8 GFlops	48×
**WRF-Chem RADM2** [11]	1 Core (3 GHz Quad-Core Xeon 5400)	1.46 GHz GTX 280	21.0944s	2.475s	8.5×
**CAM RADDEDMX** [12]	1 Core (2.6 GHz Intel Core 2 Duo)	0.65 GHz GTX 9800 GX2	0.782GFlops	10.93GFlops	14×
**WRF Microphysics Scheme** [13]	1 CPU (3.2 GHz Intel Core i7 970)	1.215 GHz GTX 590	2530ms	36.4ms	70×
**GRAPES WSM6 Scheme** [14]	1 Core (3.3 GHz Intel Core i5 3550)	1.05 GHz GTX 605	1.74s	12.4ms	140×
**PSTSWM Method** [15]	2 Microprocessors (2.8 GHz Pentium 4)	2 FPGAs (100 MHz SRC-6)	31.25ms	129ms	0.24
**Dynamic Core**					
**NIM Dynamics** [16]	1 Core (Intel Harpertown)	1.46 GHz GTX280	8.814s	0.263s	33.6×
**GRAPES Dynamics** [17]	1 Core (Intel Xeon 5500)	1.3 GHz Tesla C1060	805ms	83ms	9.7×
**CAM HOMME Dynamics** [18]	1 CPU (2.6 GHz AMD Opteron 2435)	1.15 GHz Fermi C2050	1GFlops	6.1 GFlops	6.1×
**HIRLAM Dynamics** [19]	1 Core (2.93 GHz Intel Core i7-940)	1.4 GHz GTX 480	6620ms	118.1ms	56×
**NICAM Dynamics** [20]	1280 Cores (2.9 GHz Intel Westmere-EP)	320 (CPU core + 1.1GHz M2050 GPU)	780 GFlops	2.3TFlops	3×
**Model**					
**ASUCA Model** [3]	1 Core (2.4 GHz AMD Opteron)	1 GPU (Tesla S1070, 1.44GHz)	0.355GFlops	28.4TFlops	80×
**POM Model** [21]	408 Cores (Intel E5-2670, 2.6 GHz)	4 GPUs (K20X, 2.6 GHz)	27s	27s	1 × ^†^
**Limited Area Model** [22]	12 Cores (2.7 GHz Intel Xoen X5650)	6 FPGAs (150 MHz Virtex 6 SX475T)	2235s	30s	74×

In recent years, we start to see projects that accelerate the dynamic cores (or the major computation parts of the dynamics cores) on GPU devices, such as the efforts on NIM [16], GRAPES [17], CAM-SE [18], HIRLAM [18], and NICAM [20]. Compared with the physics modules, the dynamic parts generally require communication across different grids, and are more difficult to achieve good parallel performance. For most existing projects, the achievable speedups for the dynamic cores is around 3 to 10 times when comparing GPU solutions against parallel CPU solutions (details are shown in Table 1). The only exceptions are the simplified cases that port dynamic kernels of small-scale experiments into a single GPU [16, 19], which demonstrates similar speedups to the porting of standalone physics modules.

Till now, we only see a few models that is completely ported onto the GPU platform. One example is the GPU-based acceleration of ASUCA, a next-generation high resolution mesoscale atmospheric model being developed by the Japan Meteorological Agency (JMA) [3], with an acceleration of 80-fold when compared against a single CPU core, and an excellent scalability for up to a few thousand nodes. Another example is the complete porting of the Princeton Ocean Model (POM) onto GPU devices [21], which performs a manual porting and optimization of POM onto a hybrid server with 4 GPUs, and achieves an equivalent performance to 408 CPU cores.

Compared with the various GPU-based weather or climate code projects mentioned above, the published results on FPGAs [15, 22, 23] are relatively less to be seen, and are mostly at an initial stage that evaluates the potential by porting various kernels. For example, the acceleration work [22] that is based on the reconfigurable hardware is able to provide an order of magnitude speedup through a customized design on both hardware architecture and the computation precision. Other efforts also include using the Sunway TaihuLight supercomputer [24] for large-scale atmospheric study [25, 26]. The Sunway TaihuLight contains over 40960 SW26010 many-core CPUs (over 10 million cores), and is now the most powerful supercomputer in the world with a peak performance of over 125 PFlops. Based on the new supercomputing system, the CAM [12] model has been successfully ported with a performance of 2.81 SYPD on over 1.5 million cores [25]. Another work [26] that won the 2016 ACM Gordon Bell Prize has developed a fully implicit solver for nonhydrostatic atmospheric dynamics, using more than 10 million cores to sustain an aggregate performance of 7.95 PFlops in double-precision at the 488 m horizontal resolution.

While some work listed in Table 1 demonstrated impressive performance speedups, it is also important to look at how the comparisons over the original work are implemented. For the original references, some work [11, 14, 16, 17, 19, 21] might be based on serial single-core versions. The compiler options in different work were also different. For example, Intel Fortran compiler version 10.0 with option -03 was used in the work [4] to optimize WRF WSM5. Gfortran compiler with options -O3, -ftree, -vectorize-ftree-loop-linear, and -funroll-loops was used in the work [13] to optimize WRF Microphysics Scheme. Gfortran/gcc4.3.1with option of -O3 as used in the work [14] to optimize GRAPES WSM6 Scheme. Some work [19] even used O2 option for its CPU implementation. On the contrary, some work [18] claimed to have optimized the original versions to the best for fair performance comparisons.

As for the implementations based on different accelerating systems, while a lot of optimizing efforts has been made towards the best performance improvement, issues such as the external data transfer are also important to determine the speedups. For example, some results [4, 11, 15, 16] shown in Table 1 do not involve the data transfer between CPU and corresponding accelerators. In the work [16] to optimize NIM Dynamics, if data transfer was taken into account, the speedup would drop from 33.6 to 0.74. In the work [21] to migrate POM onto GPU, the data transfer time was claimed to be well overlapped by the computing time.

In this work, we intend to provide as fair comparisons as possible, so all original references based on the CPU are carefully optimized including using multi-threading and vectorizations. Data transfer and other costs such as initializations will also be involved when counting the performance on MIC, GPU, or FPGAs.

As far as we know, this work is the first complete study on the porting and optimization of the global shallow water equation solver, which is a key component in modeling of atmospheric dynamics, across three different hybrid architectures (CPU-GPU, CPU-MIC, and CPU-FPGA). Based on our previous efforts on Tianhe-1A [27], Tianhe-2 [28], and Maxeler DFE platforms [29], this paper describes our generalized partition scheme to support various accelerator architectures, and a complete evaluation and analysis of all available accelerator technologies and their corresponding programming and porting paradigms.

## Methods

### Equations and discretizations

The global shallow-water equations (short for SWEs), which exhibit some essential characteristics of the atmosphere, are one of the most important equation sets that are selected to model the global atmosphere. Compared with the horizontal scale of the earth (around 40,000 kilometers), the thickness of the global atmosphere is only around 100 kilometers. Therefore, the SWEs take a simplified assumption of the vertical structure of the atmosphere, and are capable of describing certain large-scale phenomena in which the curvature and the complex horizontal variations of the earth can be demonstrated. Based on the above considerations, we take the SWEs as our starting point for evaluating various architectures.

**Fig 1:**
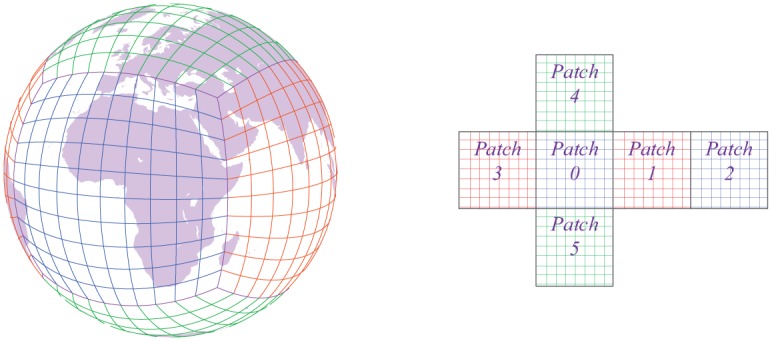
The cubed-sphere mesh (Left), and its six patches as the computational domain (Right).

The cubed-sphere mesh (the left panel of Fig 1) is selected in this work to discretize the shallow-water equations on the sphere. By mapping an inscribed cube to the surface of the sphere, the computational domain of solving the equations is the six patches from the inscribed cube (the right panel of Fig 1). We employ the cubed-sphere mesh due to the two inherent advantages as follows:

The first advantage is that, when written in local coordinates, shallow-water equations have an identical expression on the six patches:

$$\begin{array}{c} \frac{\partial Q}{\partial t}+\frac{1}{\Lambda }\frac{\partial (\Lambda F^{1})}{\partial x^{1}}+\frac{1}{\Lambda }\frac{\partial (\Lambda F^{1})}{\partial x^{2}}+S=0, \end{array}$$ (1)

where (*x*^1^, *x*^2^) ∈ [−*π*/4, *π*/4] refer to the local coordinates of a patch, *Q* refers to the prognostic variables (*h*, *hu*^1^, *hu*^2^)^*T*^, in which *h* is the thickness of the atmosphere and *hu*^1^, *hu*^2^ are the contravariant velocity components, *F*^*i*^ = *u*^*i*^ *Q* (*i* = 1, 2) is the convective flux, and *S* is the source term that can be derived from the gravity and Coriolis force.

Due to the non-orthogonality of the cubed-sphere, all additional variable coefficients introduced in Eq (1) have fixed expressions that only depend on their geometric positions; details can be found in previous works [30, 31]. The identical computing pattern in all different patches and grids makes it possible to derive a generalized partition scheme that suits various hybrid architectures. Similar results have been reported for existing atmospheric models that employ cubed-sphere meshes, such as CAM-SE [32], and atmospheric component (AM3) of the Geophysical Fluid Dynamics Laboratory (GFDL) coupled model (CM3) [33].

The second advantage is that, compared with the latitude-longitude grid, the cubed-sphere mesh removes the pole singularity issue. The avoidance of singularity problems not only improves the accuracy of the method, but also reduces the complexity of the computational schemes that we need to apply in the pole regions, which again leads to a more balanced load of computations among different grids. As for the 8 extraordinary vertices globally (at the corners of each patch), various tests have been done using benchmark test cases [34], and no accuracy degradation is observed at these weak singular points.

Suppose each patch of the cubed sphere has *N* × *N* mesh cells (the right panel of Fig 1), we can use a single vector variable to approximate all the prognostic variables within each mesh cell:

$$\begin{array}{c} Q_{ij}^{k}\left(t\right)=\frac{1}{\Lambda_{ij}^{k}}\int Q\left(t\right)dC_{ij}^{k},\;\Lambda_{ij}^{k}=\int dC_{ij}^{k}, \end{array}$$

where $C_{ij}^{k}$ refers to the mesh cell at the position of (*i*, *j*) of patch *k*, and $dC_{ij}^{k}=\Lambda dx^{1}dx^{2}$. The following semi-discrete system can be generated by integrating Eq (1) over each mesh cell.

$$\begin{array}{c} \frac{\partial X(t)}{\partial t}+L\left(X\left(t\right)\right)=0, \end{array}$$ (2)

which is integrated in time by using a second-order total variation-diminishing Runge-Kutta method (TVD-RK-2) [35] that reads

$$\begin{array}{ccc} \bar{X}\left(t^{\left(n\right)}\right) & = & X\left(t^{\left(n-1\right)}\right)-\Delta tL\left(X\left(t^{\left(n-1\right)}\right)\right),\\ X(t^{\left(n\right)}) & = & \frac{1}{2}\{X\left(t^{\left(n-1\right)}\right)+\bar{X}\left(t^{\left(n\right)}\right)\}-\frac{1}{2}\Delta tL\left(\bar{X}\left(t^{\left(n\right)}\right)\right). \end{array}$$ (3)

**Fig 2:**
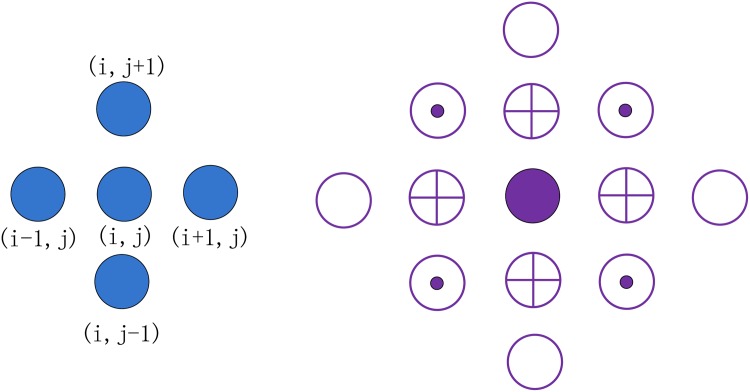
State reconstruction in cell (*i*, *j*), values in the adjacent four cells are needed (Left). The 13-point upwind stencil (Right): values of the adjacent twelve cells from different positions are needed to compute the value of the solid point in the center.

A cell-centered finite volume method is used to spatially discretize the shallow-water equations as shown in Eq (1). In each time step of the TVD-RK-2 framework there are two stencil evaluations. To be more specific, we denote $L\left(X\left(t\right)\right)=\left[L_{ij}^{k}\left(t\right)\right]$ where

$$\begin{array}{c} L_{ij}^{k}\left(t\right)=\frac{4N}{\Lambda_{ij}^{k}\pi }\int f\left(Q\left(t\right)\right)d\partial C_{ij}^{k}+S_{ij}^{k}\left(t\right), \end{array}$$ (4)

with *f* = (*F*^1^(*t*), *F*^2^(*t*)) ⋅ **n** being the normal flux. In order to calculate the integral of *f*(*Q*) on any cell edge, we use a modified Osher’s Riemann solver [36] as

$$\begin{array}{c} \int f\left(Q\right)d\partial C_{ij}^{k}\approx f_{\text{Osher}}\left(Q^{-},Q^{+}\right), \end{array}$$ (5)

where *Q*^−^, *Q*^+^ are the reconstructed states of *Q* on each cell edge from both limit sides using the value of $Q_{ij}^{k}$ on several adjacent neighbors. We use the kappa-scheme to do the state reconstruction, where 0 < *kappa* < 1. The reconstruction is formally third-order when *kappa* = 1/3 and second-order otherwise. For example, as shown in the left panel of Fig 2, two reconstructed states of *q* in the *x*^1^ direction are obtained via

$$\begin{array}{ccc} q_{i-1/2,j}^{+} & = & \frac{16}{24}q_{i,j}+\frac{1}{24}\left(q_{i,j-1}+q_{i,j+1}+3q_{i-1,j}-q_{i+1,j}\right),\\ q_{i+1/2,j}^{-} & = & \frac{16}{24}q_{i,j}+\frac{1}{24}\left(q_{i,j-1}+q_{i,j+1}+3q_{i+1,j}-q_{i-1,j}\right). \end{array}$$

When necessary, additional linear interpolation is done across the interfaces of the six patches to properly pass information without loss of accuracy and mass conservation.

Putting it together, the stencil used in the calculation of Eq (4) exhibits a diamond shape, with 13 points in total, as shown in the right panel in Fig 2. Points with different shapes demonstrate different coefficients in the stencil computing. Due to the hyperbolic nature of the SWEs, the computation of *f*_Osher_ could not be performed without a proper upwinding mechanism; i.e. “if-else” statements are used in the code to calculate stencils, which affects the computation efficiency on parallel architectures.

### General algorithm and computational challenges

Algorithm 1 shows the general workflow for solving the SWEs at each time step. For each of the six cubed-sphere patches, firstly, halos must be updated (line 2). Each patch needs to fetch the halo values from its four neighboring patches. Secondly, a linear interpolation (line 3) is carried out on the halo across patch interfaces to properly transfer halo information for stencil computations. Then we do the stencil calculation (line 4-8), which includes the computation of local coordinate based on global index *j* and *i*, and the computation of Flux variables, State Reconstruction, Reimann Solver and Source Terms (*h*, *hu*^1^, *hu*^2^).

**Algorithm 1 The General Algorithm for each stencil step**

1. **for** all the six patches **do**

2.   Halo Updating

3.   Interpolations on halos when necessary

4.  **for** all the mesh cells in each patch **do** //Upwind Stencil

5.   Compute Local Coordinate based on global index (*j*, *i*)

6.   Compute Flux, State Reconstruction, and Riemann Solver

7.   Compute Source Terms for *h*, *hu*^1^, *hu*^2^

8.  **end for**

9. **end for**

The SWEs algorithm brings serious challenges for designing efficient solutions on hybrid platforms. Halo updating and interpolations involve data communication between patches, and bring conditional statements (if…else) that have some effects on the performance of different platforms. This is particularly challenging for FPGAs due to the limited number of hardware resources. Compiler needs to deploy hardware circuit for all branches, which consumes a lot of precious hardware resources.

Moreover, although the upwind stencil from SWEs only involves 13 points (Fig 2), the computational complexity is much higher than normal stencil kernels. To compute one mesh point, we will need at least 434 ADD/SUB operations, 570 multiplications, 99 divisions, 25 square roots, 20 sine/cosine operations, and 6 tangent operations. Achieving a high level of parallelism with such a high arithmetic density is another design issue to consider when using reconfigurable platforms.

### A generalized partition scheme

#### A three-layer decomposition approach

Our study targets on scaling atmospheric simulation on supercomputers that consist of thousands of nodes and various kinds of computing resources. Therefore, one important design issue is to achieve a generalized partition scheme that can map different portions of the computational domain to different cores in the supercomputer system.

**Fig 3:**
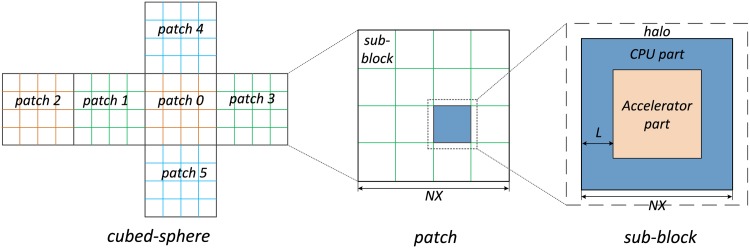
The three-layer partition scheme.

In general, our partition scheme can be divided into three different layers, as shown in Fig 3:

- At the first layer, the global computational domain is divided into the six different patches of the cubed-sphere mesh. Each patch is processed by a group of MPI processes that reside on a number of compute nodes in the supercomputer system.
- At the second layer, we further divide each patch into *N* × *N* different sub-blocks. We process each sub-block with one MPI process, which generally corresponds to one compute node in the supercomputer system. Neighboring MPI processes exchange halo information to advance the stencil updates. Meanwhile, the MPI process that corresponds to the boundary regions of the patch also needs to perform the interpolation and halo data communication between neighboring patches.
- At the third layer, we partition the sub-block into CPU region and the accelerator region, so as to keep both kinds of computing resources fully utilized in a balanced manner.

#### Balanced partition between CPUs and accelerators

To support the three different hybrid architectures (CPU-GPU, CPU-MIC, CPU-FPGA) that we target on, our scheme supports an adjustable partition between the CPUs and the accelerators within a single node. With an adjustable partition scheme, we can tune the partition scheme to achieve a balanced utilization of both CPU resources and accelerator resources. Meanwhile, the scheme also supports multiple accelerators of the same type within a single node.

**Fig 4:**
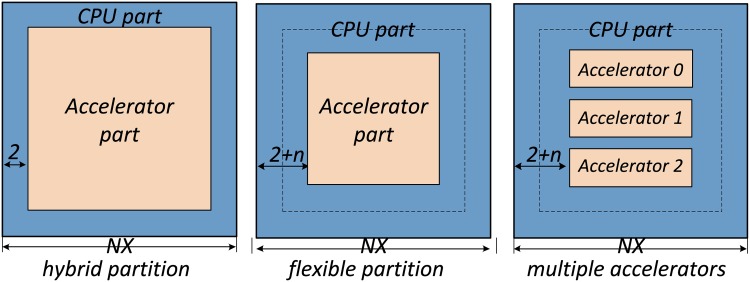
Our flexible partition scheme.

For a compute node that includes both CPUs and an accelerator, a straightforward partition scheme is shown in the left panel of Fig 4. We decompose each sub-block into the inner part that does not involve communication with neighbors and performs uniform operations on different points, and two layers of outer part that involves conditional statements, communication with neighbors, and possible interpolations when sitting on the boundary part of patches. Within each node, we employ the accelerator to process the inner part, and employ the multi-core CPUs to process the four halos.

However, as current CPUs also provide high computing power, the CPU part is finished ahead of the accelerator part in most scenarios. Therefore, to achieve a balanced utilization of both parts, we further specify a flexible CPU portion within the inner part (the central panel of Fig 4), where there is an extra part (*n* layers) being assigned to CPU. Accordingly, both CPU and accelerator finish the computations with approximately the same amount of time, with none of them idling.

For compute nodes with multiple accelerators, we employ a different strategy, shown in the right panel of Fig 4. We further decompose the inner part into a number of accelerator areas, and a number of exchange areas in between. Note that we decompose the inner part along one dimension only, so that we can achieve aligned memory access and an efficient buffering behavior for different accelerators.

**Fig 5:**
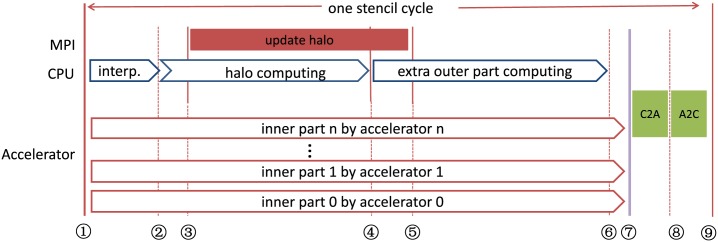
Work flow of the hybrid partition. Using *n* + 1 accelerators to process the inner part, and using CPU to process the interpolation, computing of halo and extra outer part. C2A and A2C refer to the data exchange between CPU and the accelerators. Part of the CPU time shown in the figure can be overlapped by the accelerator time.

Fig 5 demonstrates the workflow of the balanced partition, where multiple accelerators are being used. CPU and the accelerators are now working simultaneously to solve the problem, whilst the CPU task for computing and communication is hidden by the accelerator computing. A well communication-computation overlapping is obtained hereby. MPI 3.0 is employed for each hybrid system to control the process-level task. In Fig 5 we can see two types of communications. The communication between CPU and accelerators (shown as C2A and A2C) is very small and only brings slight influence on the overall performance. The communication between different computing nodes (shown as update halo) is overlapped by the computations.

Another advantage of the partition between CPU and accelerators is that by doing this, tasks to computing the inner points and outer points are separated and assigned for either CPU or accelerators parts respectively, so we no longer have the if-else branches to judge the locations. This is particularly helpful for FPGA, as otherwise compiler would have to deploy hardware circuit for all branches, which consumes a lot of precious hardware resources. As for MIC and GPU, the partition could also bring some benefits to help avoid the worst cases where GPU neighboring threads within single warp execute the conditional differently; or where MIC fails the branch prediction.

#### Workload auto-tuning mechanism

Based on the flexible partition scheme, an auto-tuning mechanism can be implemented to balance the workloads between CPU and corresponding accelerators. The key step is to dynamically track the value of *n* of Fig 3 and find the optimal one when both CPU and accelerator finish the computations with approximately the same amount of time. We can write a script (*e.g.* using bash or shell) that automatically compiles and executes the code based on different values of *n*, and record the corresponding time of CPU computation and accelerator computation, respectively. Such method is easy to implement based on CPU, MIC, and GPU as the compiling steps for those architectures do not cost too much time. However, compiling FPGA every time when *n* is updated is unrealistic to implement, as hardware compiling to deploy new chip circuit generally requires a long period of time (at least two hours in our cases). Therefore, instead of compiling FPGA frequently, we use the performance prediction model we proposed in previous work [37] to estimate the time of FPGA computation and narrow down the value of *n* to a small range. In this way, a lot of time is saved. The performance prediction model is proposed according to the fact that when a highly-efficient pipeline flow is built on the FPGA chip, there will be one output at every physical cycle. So when such information as the size of data and the frequency of FPGA chip is given, the performance model can be formulated and predicted.

#### “Pipe-flow” communication

While the cubed-sphere mesh already achieves a balanced mapping of computation loads to various nodes, we also propose a carefully designed communication strategy, which is called the “pipe-flow” scheme, to conduct a balanced message passing, and a balanced utilization of the entire network.

**Fig 6:**
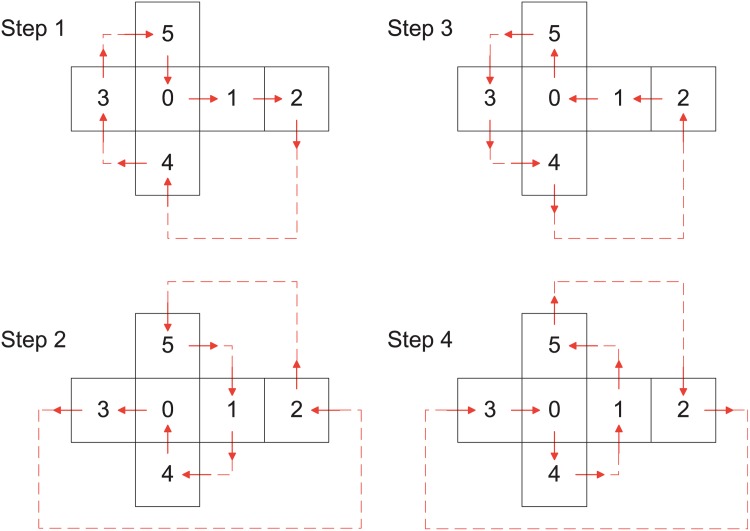
The “pipe-flow” communication scheme with four steps. Arrows indicate the directions that data flows the six patches. At each step, each patch only has one data in (MPI receive) and one data out (MPI send), leading to a better load balance systematically.

As shown in Fig 6, the neighboring patches exchange the information in four different steps. Communications are performed like a flow going through a closed loop covering the six patches of the cubed-sphere, with inlet/outlet directions of the flow on each patch. Within the patch, we take a similar approach to managing the communication flow between different sub-blocks. We are using a pair of neighboring communication functions (such as VecScatterBegin/End) from the framework of PETSc (Portable Extensible Toolkit for Scientific computation [38]) to finish the data exchange. More detailed information about the “pipe-flow” scheme can be found in previous work [27].

### Solving SWEs on a CPU-MIC platform

#### The Tianhe-2 supercomputer

The Tianhe-2 supercomputer [9] is one of the most powerful supercomputers of the world (No.2 in the latest TOP 500 list), and is installed in the National Supercomputing Center in Guangzhou. The peak performance of Tianhe-2 is 54.9 Pflops, with a sustained LINPACK performance of 33.9 Pflops. Tianhe-2 consists of 16,000 computing nodes, each of which is equipped with two Intel Xeon E5-2692 processors, 3 Intel Xeon Phi 31S1P accelerators, and 64GB memory.

**Fig 7:**
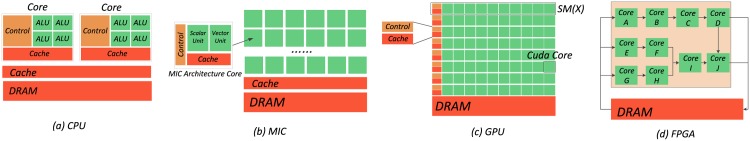
The hardware architecture of the multi-core CPU, MIC, many-core GPU, and reconfigurable FPGA.

The Intel Xeon Phi accelerator is manufactured based on the Intel Many Integrated Core (MIC) architecture, shown in Fig 7(b). Each Intel Xeon Phi 31S1P accelerator installed in Tianhe-2 has 57 cores running at 1.1GHz and 8GB on-board GDDR5 memory all connected by a high performance bidirectional ring. While GPU and MIC are both called many-core architectures, MIC takes a different approach to stacking more computing resources into one chip. To make the porting of CPU code less complicated, the MIC architecture is using a previous generation Pentium core to keep the compatibility of x86 code. Therefore, each MIC core is more like a simplified CPU core. As MIC core does not support out-of-order execution, 4-way hyper-threading support is added to help hide memory and multi-cycle instruction latency. More details about MIC architecture can be found in related article [39].

#### Designing and optimizing the SWE solver for MICs

We carefully schedule the various computation parts, and communication parts for both CPUs and MICs. The complexity of the flow increases as we have an increased number of accelerators. One difference from our work on Tianhe-1A (introduced in the following section) is the partition scheme of the computational domain. In the CPU-MIC case, as we have three MIC accelerators, we decompose the domain according to the right panel of Fig 4.

As MIC shares common features with CPU in both the programming part and the architecture, similarly, we use OpenMP and SIMD vectorization to achieve most of the parallel performance. Considering the 4-way hyper-threading support, in most cases, setting the number of threads to four times of the number of cores gives the best performance. Through adding −*simd* option to the compiler, the AVX SIMD instructions can be generated automatically on the Xoen Phi of Tianhe-2.

However, as the MIC core is a simplified design that removes a number of features in CPUs, we still need to tune the code carefully for reasonable performance. Our first challenge is that the MIC architecture does not include hardware support for trigonometric functions needed for the non-linear parts. Therefore, the 6 tangent (TAN) functions lead to long latency and performance degrade. In the 6 TAN computations, we can compute and store one x-related and one y-related TAN values during initialization. For the other 4 TANs, we use the formulation $\tan\left(A+B\right)=\frac{\tan(A)+\tan(B)}{1-\tan(A)\tan(B)}$ to calculate them. In this way, we replace the 6 TAN computations by 4 ADD, 4 SUB, 4 MUL, 4 DIV computations, and 2 memory accesses. We apply a similar strength reduction strategy to other long-latency instructions, such as SQRT and DIV. Overall, the strength reduction strategy is able to contribute 10% improvement to the time-to-solution.

### Solving SWEs on a CPU-GPU platform

#### The Tianhe-1A supercomputer

As a petascale heterogeneous supercomputer, Tianhe-1A [8] includes 7,168 computing nodes with a peak performance of 4.7 Pflops. Each node consists of two six-core Intel X5670 CPUs with 32GB local memory and one NVIDIA Fermi M2050 GPU with 3GB onboard memory.

Originally designed for graphical functions, the GPU architecture demonstrates a lot of similar features to the vector machines that dominate the HPC community in the 80s and 90s. As shown in Fig 7(c), the computing power is provided from a number of streaming multiprocessors (SM). Each SM includes a large number of CUDA cores (32 CUDA cores for the SM in Fermi cards of Tianhe-1A, and 192 CUDA cores for the SMX in the most recent Kepler GPU cards) that are scheduled for execution in a vector-like way. The CUDA cores within each SM share the same cache and shared memory modules. The data is fed to the CUDA cores through 16 load/store units in the SM. The cache and shared memory module provides an extremely high bandwidth through a group of 32 memory banks. In terms of programming, the computation is performed through a large number (up to millions) of light-weight threads, which are organized as warps (each warp consists of 32 threads). Therefore, to achieve a high performance on GPU devices, we shall organize both the computation and the memory access in a vectorized way. More details about GPU architecture can be found in related article [40].

#### Designing and optimizing the SWE solver for CPUs and GPUs

As mentioned before, we generally allocate operations that are not suitable for vectorized accelerators to the CPUs, such as the conditional statements and communication needed for the halo regions.

On CPU, we use OpenMP to spawn different threads to process different rows and columns. Common stencil code optimization techniques, such as array padding and in-loop SIMD vectorization [41] are used. As memory access is a major factor that limits the performance, we set the number of OpenMP threads to be twice of the number of CPU cores to hide a part of the memory access latency.

The method to parallelize computation on the GPU is quite different. Using the two-layer block and grid thread hierarchy of CUDA, we compute each single grid point using a different CUDA thread. Applying a similar idea to the 3D stencil work [42], we use the shared memory as the common buffer for sharing neighboring points in the same thread block.

Another optimization strategy we take is the on-the-fly preparation of auxiliary vectors. Several variable coefficients such as the tensor terms, the Coriolis source term and the topographic term, are needed in the evaluation of the nonlinear stencils. These coefficients are only dependent on their geometry positions and remain unchanged during the whole calculation. Therefore a common practice is to compute and store them as auxiliary vectors for reuse. However, as memory bandwidth becomes the major bottleneck in the solving process of SWEs, we compute 18 out of the 20 auxiliary vectors in run time. Such a strategy gains performance benefits for both CPU and GPU architectures, and is able to decrease the computational time by 10% and 58% for CPU and GPU, respectively.

### Solving SWEs on a CPU-FPGA platform

#### The Maxeler DFE architecture

The exploration of FPGA accelerators is performed on the Maxeler FPGA platform [43]. The MaxNode is a server-class HPC system, and contains 12 Intel Xeon CPU cores and 4 DFEs. Each DFE contains one Virtex-6 SX475T FPGA and 24GB on-board memory (DRAM).

Different from multi-core CPU and many-core GPU, MIC architectures, the FPGA-based DFE is a completely customizable hardware that can be programmed into an arbitrary form of circuit. Therefore, in most scientific applications, an FPGA-based design takes a data-flow oriented approach to performing the computation. As shown in Fig 7(d), the algorithm, i.e., the operations that we want to perform, is mapped into circuit units along the path from the input ports to output ports. The computation gets performed as the data streams through the circuit units in one pass.

#### Designing and optimizing the SWE solver for FPGAs

While the reconfigurable FPGAs provide the advantage of complete architectural flexibility, the disadvantage is the lack of inherent hardware support for floating-point arithmetic. Therefore, implementing a double-precision SWE kernel on the FPGA can be extremely resource demanding. In our case, a straightforward implementation of the entire 1,000 floating-point operations consumes around 2.5 times of the available resources that one DFE can provide.

Our first solution is to decompose the SWE solver into three different parts that reside on three different DFEs (denoted as **double-precision** design). We perform the kernel decomposition with the following two metrics: (1) minimized inter-communications between the different sub-kernels, so as to avoid that the interlink between different FPGAs becomes the performance bottleneck; (2) balanced resource costs of different sub-kernels, so that we can achieve a balanced utilization of different DFEs. By decomposing the entire solver into three DFEs (DFE1: the left and right directions of the three major steps: Flux computation, State Reconstruction, and Riemann Solver; DFE2: the top and bottom directions of the three major steps; DFF3: the Source Terms computing module), we manage to fit the SWE solver into three DFEs within one MaxNode.

Although we manage to build an efficient pipelined design of the solver, the relatively low bandwidth between different DFEs severely limits the performance of the entire solution. To further improve the performance, we consider the strategy of performing the computation in a mixed-precision (denoted as **mixed-precision** design). We firstly perform a range analysis to track the dynamic range of different intermediate variables. Then we divide the solver into the parts with a small dynamic range (such as the State Reconstruction part), where we can use fixed-point numbers; and the parts with a wide dynamic range, where we continue to use floating-point numbers. After ensuring that the range is covered, we then iterate through a different number of precision bits, and perform numerical simulations to identify the minimum precision that can still guarantee accurate-enough simulation results. In a final design, we are using fixed(2, 38) (fixed-point numbers with 2-bit integer part and 38-bit fractional part) and float(8, 32) (floating-point numbers with 8-bit exponent and 32-bit mantissa), which keeps a relative error within 2% and a resource cost within 80% of one DFE. To prevent the bandwidth from becoming the bottleneck of the overall performance, we put all the input data onto the 24 GB on-board memory (DRAM).

More detailed description about our FPGA-based design can be found in previous work [29].

## Results and discussions

### Scaling on Tianhe-1A and Tianhe-2

This part demonstrates the performance scalings on the two heterogeneous supercomputers, Tianhe-1A and Tianhe-2.

The numerical solutions are validated based on the comparison with a standard benchmark [44], *i.e.*, zonal flow over an isolated mountain. The simulated results on MIC and GPU are in good agreement with the standard in the benchmark [44]. Note that due to the truncation error, the relative error that is near to machine epsilon (around 10^−14^ in double precision) is a tolerance in our case. Corresponding surface level distribution of MIC and GPU is provided in our previous works [27, 28]. For FPGA solution, as mixed precision brings loss of accuracy, the conservation property is relaxed to 10^−11^ [45]. Related work [46, 47] demonstrates the feasibility of employing mixed-precision while ensuring a reasonable simulation accuracy.

**Fig 8:**
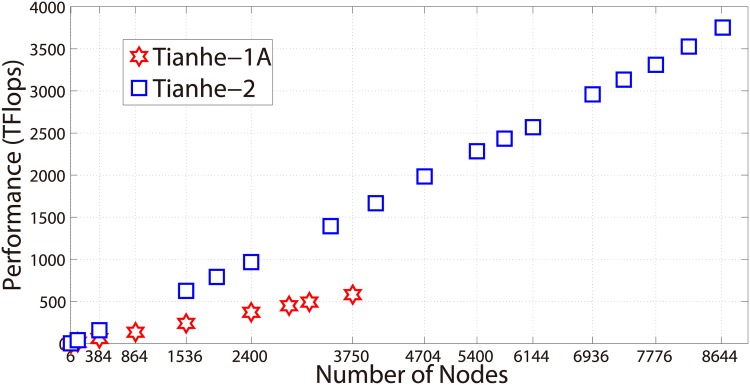
Weak scaling performance on Tianhe-1A and Tianhe-2.

In the weak-scaling tests, we fix the mesh size of each sub-block to be 1024 × 1024 and 4096 × 4096 for Tianhe-1A and Tianhe-2, respectively, and gradually increase the number of computing nodes. The performance results of the weak-scale tests are shown in Fig 8. On the Tianhe-1A supercomputer, the number of computing nodes is increased from 6 to 3750, and the total number of unknowns is raised from 18.8 millions to nearly 12 billion. An aggregate double-precision performance of 581 TFlops (around 23.5% of the peak) is achieved at 3750 nodes configuration. On the Tianhe-2 supercomputer, the computing scale is increased from 6 nodes to 8664 nodes. Within each node, the three MICs and the 24 CPU cores are fully exploited. In the largest scale, about 1.69 million cores are employed and the the total number of unknowns reaches up to 436 billion.

The tests on both supercomputers have exhibited nearly linear performance scalings, which suggests that the generalized partition scheme we proposed manages to achieve good computation-communication overlapping, with most of the overhead caused by the increased amount of communication being hidden by the computation.

**Fig 9:**
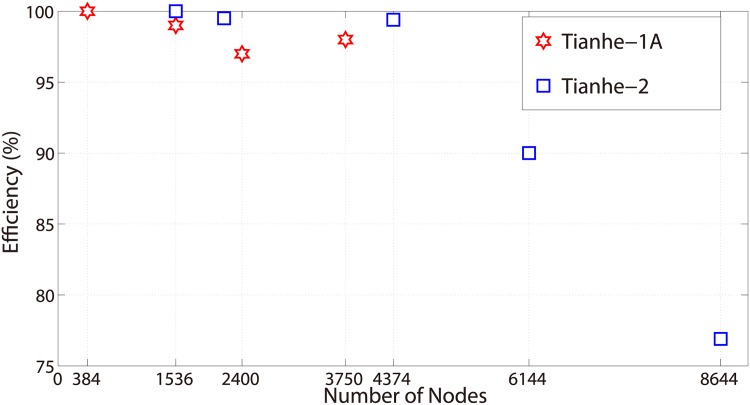
Strong scaling efficiency on Tianhe-1A and Tianhe-2.

In the strong-scaling tests, we set the total mesh size to be 25200 × 25200 × 6 and 65536 × 65536 × 6 on Tianhe-1A and Tianhe-2, respectively, and gradually increase the number of computing nodes. In the designs for both supercomputers, we calculate the averaged mesh size on each computing node and assign a proper portion of the outer layers to CPUs so that optimal performance can be achieved. The parallel efficiencies of the strong scaling tests are shown in Fig 9. As for the Tianhe-1A supercomputer, we can find out that nearly ideal strong scaling efficiency is sustained when increasing the number of computing nodes. There is only a very slight loss of scalability due in part to the non-overlapping communications, such as the G2C and C2G parts shown in Fig 5. The performance scaling on Tianhe-2 also demonstrates nearly ideal efficiency when the number of nodes is increased from 1536 to 4374. However, it decreases to 90.0% when using 6144 nodes and further down to 76.9% when using 8644 nodes. Such degradation in efficiency is acceptable as the working load for each node is too small to scale at a higher node count.

### Large-scale simulation and validation

**Fig 10:**
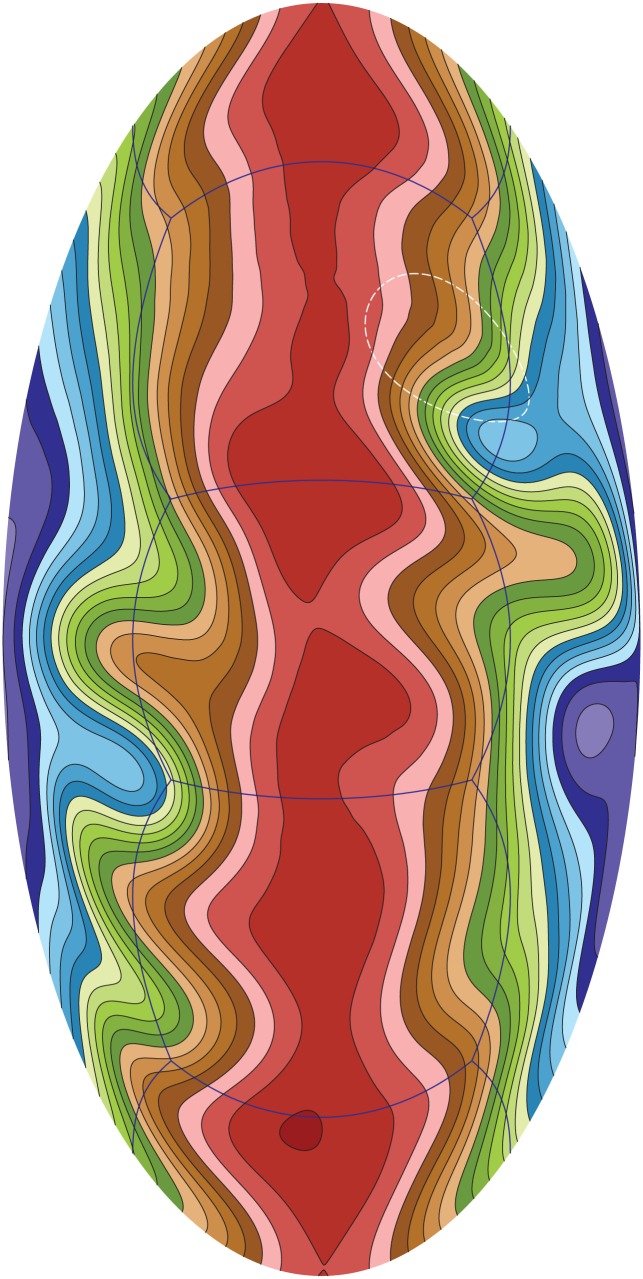
Surface level distribution of the atmosphere at day 15 on Tianhe-1A based on the model test [34]. The conical mountain is outlined by the dotted circle.

Fig 10 shows the simulation results on Tianhe-1A based on a model problem, zonal flow over an isolated mountain, which comes from the standard benchmark test set [34]. A geostrophically steady-state flow impinges from west to east over a compactly supported mountain of conical shape. In the figure, the surface level distribution of the atmosphere at day 15 in the isolated mountain test using 10240 × 10240 × 6 cubed-sphere mesh (around 1 km resolution) is demonstrated. 1536 of the CPU-GPU computing node of Tianhe-1A are used. The result is in good agreement to the published results [44].

**Fig 11:**
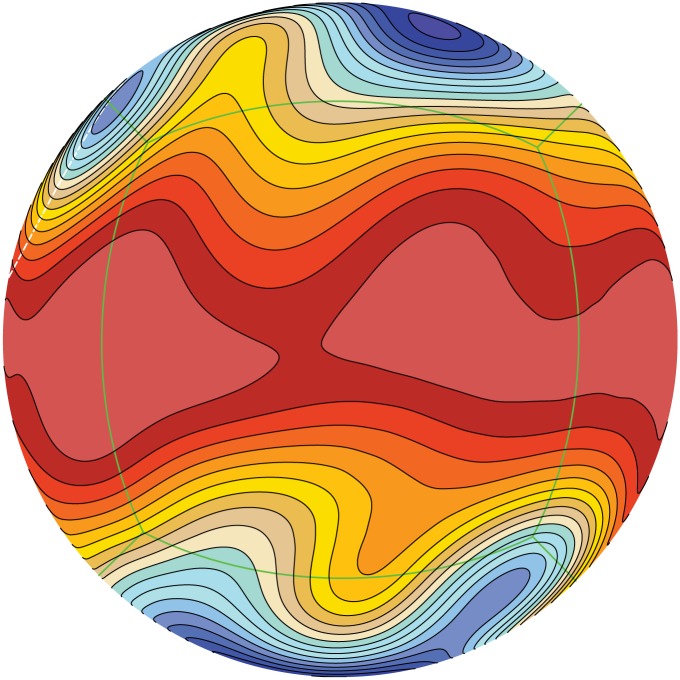
Surface level distribution of the atmosphere at day 15 on Tianhe-2 based on the model test [34].

Simulation using the same benchmark test (zonal flow over an isolated mountain) is conduct on Tianhe-2 using 1536 of the CPU-MIC computing node of Tianhe-2. The result is also in good agreement to published results [44]. In this simulation we demonstrate in Fig 11 a contour plot of the surface level distribution (*i.e., h + b*) at day 15. The well-developed Rossby-type gravity wave can be clearly seen in the figure.

Details to validate our DFE-based algorithms can be found in previous work [29, 45], which shows an acceptable discrepancy caused by the reduced data precision. More details to prove the feasibility of using mixed-precision approach can be found in some related works [46–49].

### Performance speedup and power efficiency

Table 2 demonstrates the comparisons of single-chip performance among different chips we have chosen in this work. Compared with single 6-core X5670 CPU chip, the single-chip performances of SWEs on 12-core CPU, GPU, MIC and FPGA can achieve the speedups of 2×, 12×, 17×, and 75× (mixed-precision design), respectively. The double-precision FPGA design is not discussed here, as it requires at least three chips to accomplish. Compared to the theoretical double-precision performance, the computing efficiency of 25% and 31% are achieved for Xeon Phi 31S1P and Fermi M2050, respectively. FPGA is employing a data flow computing model to implement the computations. In our case, as memory bandwidth is not becoming the bottleneck, we can have an output at every physical cycle of the FPGA chip and the computing efficiency is extremely high (95%). The slight loss of efficiency mainly comes from the initial stage to feed the first few parts of data through the pipeline. Compared with the performance benefits on CPU, OpenMP multithreading can bring a more significant performance benefits on MIC due to the surge in the number of computing cores. In our case, using OpenMP can bring a performance speedup of 14 and 120 on CPU and MIC, respectively.

**Table 2:** Single-chip performance comparison.

chip	performance (GFlops)	speedup
X5670 CPU (6 cores)	12	1
E5 2692 CPU (12 cores)	24	2×
Fermi C2050 GPU	140	12×
Xeon Phi 31S1P MIC	200	17×
Virtex-6 FPGA (mixed-precision)	900	75×

In addition, Table 3 demonstrates the comparisons of single-node performance among different computing nodes. As there are more than one accelerators inside each Tianhe-2 and Maxeler nodes, we illustrate the performance results based on different number of MICs or DFE cards. On a Tianhe-1A node, the performance using one GPU accelerator and twelve CPU cores can achieve a speedup of 7.6 × over the two 6-core CPU design. On a Tianhe-2 node, the pure CPU design using two 12-core Xeon E5 2697 CPUs can achieve a speedup of 2.2× over the CPU design of Tianhe-1A. The speedups further increase to 11.6×, 16.7×, and 19.3×, when further involving single, two and three MIC cards inside Tianhe-2 node. As for the Maxeler node, the performances using one and four DFEs (mixed-precision versions) are 46 and 152 times better than the baseline CPU design. The double-precision FPGA design is even slower than a pure CPU version, due to the limit of the bandwidth among different FPGA cards.

**Table 3:** Single-node performance comparison.

Configuration	performance (GFlops)	speedup
Tianhe-1A		
CPU (12 cores)	20.7	1
CPU + GPU	158	7.6×
Tianhe-2		
CPU (24 cores)	46	2.2×
CPU + MIC	240	11.6×
CPU + 2 MIC	346	16.7×
CPU + 3 MICs	400	19.3
Maxeler		
CPU (12 cores)	20.7	1
CPU + 3DFEs (double precision)	14	0.7×
CPU + DFE (mixed precision)	956	46×
CPU + 4 DFEs (mixed precision)	3156	152×

Table 4 further shows the power efficiency (evaluated as performance per watt). The power is measured using a power meter attached to various nodes. As each Tianhe-2 node contains three MIC co-processors and two CPU processors, it generally consumes more significant power and becomes the worst one in terms of power efficiency. Comparably, the Maxeler node and the Tianhe-1A node are 12.4 times, and 1.2 times more power efficient, respectively. Due to the lower clock frequency of the FPGA chip and the high computing efficiency of the pipeline design, FPGA-based Maxeler node provides the best power efficiency.

**Table 4:** Power efficiency of different supercomputer nodes.

	power (watt)	efficiency (GFlops/watt)	power efficiency
Tianhe-1A node	360	0.6	1.2×
Tianhe-2 node	815	0.49	1
Maxeler node	514	6.1	12.4×

### Programming paradigms

#### Programming languages

While all the three heterogeneous architectures (CPU + GPU, CPU + MIC, and CPU + FPGA) require additional programming efforts, the level of complexity and difficulty varies.

The MIC accelerator was designed to provide the best porting experience by keeping the compatibility through using a previous Intel CPU core architecture. In addition, the MIC platform shares almost the same programming, compilation, and tuning tools as the Intel CPU platforms. Therefore, in most cases, MIC shares the same kernel code as CPU, and the users only need to add OpenMP-like directives to offload the corresponding tasks to the MIC coprocessor.

The GPU accelerator is supported by two different programming approaches. One is OpenACC [50], which is a similar directive approach to OpenMP. The other one is the CUDA programming model [40], which maps the parallel computing operations into millions of lightweight threads. The threads are organized as two levels. The top level is a thread grid that can include a maximum of 65,532 thread blocks. At the second level, each thread block consists of a maximum of 512 to 1,024 concurrent threads. While OpenACC minimizes the required porting efforts, the CUDA model is used in most cases to achieve the best efficiency. When using CUDA, in addition to the codes that offloads the corresponding data and computation to the GPU devices, the users also need to write the CUDA C or CUDA Fortran descriptions of the threading hierarchy and the operations performed in each of the threads.

In the case of the FPGA accelerator, the Maxeler DFE used in our study is supported by the MaxCompiler programming platform. The users can use a high-level MaxJ (an extension of Java that includes hardware-related features) to describe the dataflow design and the configuration of the interface between the CPU host and the DFE. To enable a CPU + FPGA hybrid design, the users need to write three different parts: part one initializes the offloading of the data and the computation to the FPGA; part two describes the interfaces between the host memory and the DFE memory (how the data flows between the CPU and the DFE); part three describes the dataflow computing kernel on the DFE.

#### Code sample comparison

To provide a more concrete picture we list the code samples for CPU, MIC, GPU, and DFE platforms in Algorithms 2–5. For brevity, we only show a small portion that computes certain intermediate variables.

**Algorithm 2 CPU Code Sample**

//*xh and yh are the input and output propagation variables*

1: **void** Stencil_Compute_CPU(**double** yh[NY][NY], **double** xh[NX][NX]) {

2: #pragma omp parallel **for** num_threads(num)

3:  **for** (j = y0; j <ny; j++) {

4:   **for** (i = x0; i < nx; i++) {

5:    qL = (2/3.)∗ (xh[j][i − 1]) + (3.∗(xh[j][i])—(xh[j][i − 2]))/8 + ((xh[j + 1][i − 1]) + (xh[j − 1][i − 1]))/24.;

6:    qR = (2/3.)∗ (xh[j][i]) + (3.∗(xh[j][i + 1]) − (xh[j][i − 1]))/8 + ((xh[j + 1][i]) + (xh[j − 1][i]))/24.;

  //*hereafter omitting later steps to compute* the propagation output yh, yhu, yhv.

**Algorithm 3 CPU + MIC Code Sample**

//*MIC is to compute the inner area, while CPU is to compute the outer area*

1: __attribute__((target(mic))) **double** xh[NX][NX], yh[NY][NY];

2: #pragma omp section {

3: #pragma offload target(mic:0) nocopy(xh, yh) {

4:   Stencil_Compute_MIC(yh, xh);

6:   }

7: }

8: #pragma omp section {

9:   Stencil_Compute_CPU(yh, xh);

10: }

11: __attribute__((target(mic))) **void** Stencil_Compute_MIC(**double** yh[NY][NY], **double** xh[NX][NX]) {

12: #pragma omp parallel **for** num_threads(num)

13:  **for** (j = y0 + 2; j <ny − 2; j++) {

14:   **for** (i = x0 + 2; i < nx − 2; i++) {

15:    qL = (2/3.)∗ (xh[j][i − 1]) + (3.∗(xh[j][i])—(xh[j][i − 2]))/8 + ((xh[j + 1][i − 1]) + (xh[j − 1][i − 1]))/24.;

16:    qR = (2/3.)∗ (xh[j][i]) + (3.∗(xh[j][i + 1]) − (xh[j][i − 1]))/8 + ((xh[j + 1][i]) + (xh[j − 1][i]))/24.;

  //*hereafter omitting later steps to compute the* propagation output yh, yhu, yhv.

**Algorithm 4 CPU + GPU Code Sample**

1: cudaMalloc(&dyh, **size of (double)**∗(NY)∗(NY));

2: cudaMalloc(&dxh, **size of (double)**∗(NX)∗(NX));

3: cudaMemcpy(dxh, buffer_xh, **size of** (ActiveField)∗(NX)∗(NX), cudaMemcpyHostToDevice);

//*Hybrid working mechanism with GPU doing the inner-part stencil computation*

4: #pragma omp section {

5: dim3 dimGrid((NX)/TX, (NX)/TX);

6: dim3 dimBlock(TX, TX);

7: Stencil_Compute_GPU<<<dimGrid, dimBlock>>>(dyh, dxh);

8: cudaThreadSynchronize();

9: cudaMemcpy(buffer_yh, dyh, **size of(double)**∗NY∗NY, cudaMemcpyDeviceToHost);

10:  }

11:  #pragma omp section {

12:  Stencil_Compute_CPU(yh, xh);

13:  }

//*CUDA Kernel, define idx(j, i) to be (j ∗ TX + i)*

14: __global__ **void** Stencil_Compute_GPU(**double** ∗dyh, **double** ∗dxh) {

15: __shared__ sm[(TX + 4)∗(TX + 4)];

16:   i = blockIdx.x∗blockDim.x+threadIdx.x;

17:   j = blockIdx.y∗blockDim.y+threadIdx.y;

18:   sm[threadIdx.y∗TX + threadIdx.x] = dxh[j∗NX + i]

19:   **if** (i > = x0 + 2 && i < nx − 2 && j > = x0 + 2 && j < nx − 2) {

20:    qL = (2/3.)∗(sm[idx(j, i − 1)]) + (3∗(sm[idx(j, i)])—(sm[idx(j, i − 2)]))/8 + ((sm[idx(j + 1, i − 1)]) + (sm[idx(j − 1, i − 1)]))/24;

21:    qR = (2/3.)∗(sm[idx(j, i)]) + (3∗(sm[idx(j, i + 1)])—(sm[idx(j, i − 1)]))/8 + ((sm[idx(j + 1, i)]) + (sm[idx(j − 1, i)]))/24;

  //*hereafter omitting later steps to compute the* propagation output yh, yhu, yhv.

**Algorithm 5 CPU + FPGA Code Sample**

//*part1: in host.c*

1: #pragma omp section {

2: **const int** size = NX ∗NX;

3: **int** sizeBytes = size ∗ **size of (double)**;

4: Stencil_Compute_writeLMem(0, sizeBytes, xh);

5: Stencil_Compute(size, yh);

6: }

7: #pragma omp section {

8: Stencil_Compute_Outer(yh, xh);

9: }

//*part2: in manager.java*

1: package Stencil_Compute;

2: import manager packages;

3: public class Stencil_ComputeManager {…}

//*part3: in kernel.java*

1: package Stencil_Compute;

2: import kernel packages;

3: class Stencil_ComputeKernel extends Kernel {

4:  private **static** final DFEType type = dfeFloat(11, 53);

5:  protected Stencil_ComputeKernel(KernelParameters parameters) {

6:  super(parameters);

7:   DFEVar xh = io.input(“xh”, dfeFloat(11, 53));

  //*type casting: from double-precision to reduced precision*:

8:   DFEVar dxh = xh.cast(dfeFloat(8, 32));

9:   DFEVar qL = (2/3.)∗ (stream.offset(dxh, −1)) + (3.∗(dxh))—(stream.offset(dxh, −2))/8 + ((stream.offset(dxh, 1028 − 1)) + (stream.offset(dxh, −1028 − 1)))/24.;

10:  DFEVar qR = (2/3.)∗ (dxh) + (3.∗(stream.offset(dxh, 1))—(stream.offset(dxh, −1)))/8 + ((stream.offset(dxh, 1028)) + (stream.offset(dxh, −1028)))/24.;

 //*hereafter omitting later steps to compute the* propagation output yh, yhu, yhv.

As demonstrated in Algorithms 2 and 3, CPU and MIC are sharing the same programming model. The OpenMP *omp parallel for* directives are used on both CPU and MIC platforms to achieve multi-threading parallelism, and parameter *num* is used to control the number of threads being employed. The *section* and *offload* directives are used to distribute the computation tasks between CPU and MIC.

For the CPU+GPU code sample in Algorithm 4, similarly, we use OpenMP *section* to allocate different threads for performing computation on CPU and managing the GPU device. In old versions of CUDA, we need to explicitly call *cudaMalloc* (line 1, 2) and *cudaMemcpy* (line 3) functions to move the data from the CPU memory to the GPU memory. In contrast, for the MIC platform, the users only need to add certain specifiers to indicate that the array would be transferred to and processed by the MIC accelerator. In the most recent CUDA 6 interface, such explicit memory function calls can also be avoided by using the unified memory space feature. The parallel computation is performed by calling the kernel function using the grid and block thread configuration (line 7 in Algorithm 5). Within the kernel function, we describe the operations for each lightweight thread, and use the grid and block index to refer to the data items that correspond to the current thread. The array with the *__shared__* specifier is the fast buffer stored in the shared memory and shared among the threads in the same thread block.

As mentioned above, the CPU+FPGA code consists of three different parts. The first part that locates in file *host.c* is written in C format and is used to load *xh* onto the FPGA on-board memory (line 4), as well as to call the Kernel with *size* stream cycles (line 5). The Manager defined in part 2 is to connect the two streams *xh, yh* with the on-board memory and the Kernel. The third part illustrates the Kernel that executes the stencil computation in a dataflow approach, with two important hardware-related features. One is the *stream.offset* function which allows the user to access different locations of the data stream, and builds up corresponding data buffers behind the scene. The other one is the *DFEtype* attribute of each hardware variable. By specifying different data types for different variables (*DFEfloat* and *DFEfix*), we manage to achieve mixed-precision hardware designs.

Obviously, we do not have enough space to cover all the programming details on these different heterogeneous platforms. In Table 5, we provide an estimation about the programming and optimization difficulties using the number of coding lines as metrics. Table 5 first summarizes the total number of lines for the CPU original program, and then listed the additional lines required to be programed for each porting.

**Table 5:** The number of code lines on different platforms.

platform	CPU	GPU	MIC	FPGA
original version	969	−	−	−
naive porting	−	+96	+10	+644
optimized porting	−	+90	+80	+115
porting difficulty	−	medium	low	high

Compared with the original CPU program with 969 code lines, the porting on MIC is obviously the least difficult to implement. A naive poring only requires 10 additional lines, mainly some extra declarations. However, if we want to observe a satisfying performance improvement, another 80 lines have to be added to achieve prefetch and SIMD vectorization. The difficulty for GPU stands in the middle place. A direct porting additionally requires 96 lines, while another 90 lines are necessary to employ shared memory techniques. DFE programing is the most difficult compared with the other two counterparts, as 644 lines are needed for a native porting and another 115 lines to involve mixed-precision arithmetic.

## Conclusions

This work provides evaluation on the potential of various state-of-the-art accelerator architectures (GPU, MIC, FPGA) for performing global atmospheric simulation. A set of optimization techniques are utilized for each type of accelerator to achieve significant performance speedups. We demonstrate a highly scalable framework, with a generalized partition scheme that is capable of integrating both processor and accelerators into a highly-efficient atmospheric solving system. Based on Tianhe-1A and Tianhe-2 heterogeneous supercomputers, we investigate the scaling of the algorithm and achieve almost linear performance improvement with the increase of the number of nodes. In addition, the programming paradigms of different heterogeneous systems are also discussed to identify the cost of porting and optimizing the code.

As far as we know, this work is the first complete study on the porting and optimization of the global shallow water equation solver across three different hybrid architectures (CPU-GPU, CPU-MIC, and CPU-FPGA). Our study demonstrates promising performance benefits of running the global atmospheric simulation on heterogeneous platforms. In terms of the programming cost, the paradigms for using heterogeneous accelerators are also getting close to the level that can enable domain programmers on these platforms.

In our future work, we would extend our current work to 3D Euler solvers on these heterogeneous supercomputers, which would later evolve into a highly efficient framework for performing global atmospheric simulations.
